# Novel View on Umbilical Cord Blood and Maternal Peripheral Blood—an Evidence for an Increase in the Number of Circulating Stem Cells on Both Sides of the Fetal–Maternal Circulation Barrier

**DOI:** 10.1007/s12015-017-9763-z

**Published:** 2017-08-29

**Authors:** Katarzyna Sielatycka, Agata Poniewierska-Baran, Karolina Nurek, Andrzej Torbé, Mariusz Z. Ratajczak

**Affiliations:** 1 Department of Physiology, Pomeranian Medical University, Szczecin, Poland; 2 Department of Physiology, Faculty of Biology, University of Szczecin, Felczaka 3c, 71-412 Szczecin, Poland; 3 Department of Immunology, Faculty of Biology, University of Szczecin, Felczaka 3c, 71-412 Szczecin, Poland; 4 Department of Obstetrics and Gynecology, Pomeranian Medical University, Szczecin, Poland; 5 Stem Cell Institute at James Graham Brown Cancer Center, University of Louisville, 500 S. Floyd Street, Rm. 107, Louisville, KY 40202 USA; 6 Department of Regenerative Medicine, Medical University of Warsaw, Warsaw, Poland

**Keywords:** HSCs, MSCs, EPCs, VSELs, Stem cell mobilization, Umbilical crod blood, Maternal blood

## Abstract

Umbilical cord blood (UCB) is a rich source of stem cells, including hematopoietic stem cells (HSCs), mesenchymal stem cells (MSCs), endothelial progenitors cells (EPCs), and very small embryonic-like stem cells (VSELs). These cells most likely are mobilized into UCB in response to hypoxia and delivery stress. We have hypothesized that they may play a role in repairing certain tissue/organ injuries that occur in the newborn child after delivery. Here we asked whether delivery also mobilizes stem cells into maternal blood, as the mother also experiences hypoxia and several types of internal tissue injuries, particularly in the reproductive tract. We observed that the number of HSCs, MSCs, EPCs, and VSELs increases in maternal blood at 24 h after physiological delivery (*n* = 17). Based on this observation, we propose that delivery stress is associated with an increase in the number of circulating stem cells, not only on the fetal side but also on the maternal side of the fetal–maternal circulatory barrier.

## Introduction

Umbilical cord blood (UCB) is a rich source of various stem cells that may be employed for hematopoietic transplantations as well as other applications in regenerative medicine. The first successful hematopoietic transplantation employing UCB hematopoietic stem cells (HSCs) was reported in 1988 [1]. Currently, close to 40,000 UCB procedures have been performed worldwide, and close to 800,000 UCB units are stored in private and public UCB banks [2]. Potential application of other types of UCB stem cells, such as mesenchymal stem cells (MSCs), endothelial progenitor cells (EPCs), and very small embryonic-like stem cells (VSELs), are currently being studied in animal models and in early-stage clinical trials to apply these unique cells to treating cardiovascular diseases, neurological deficits, liver diseases, immune system diseases, diabetes, and lung and kidney injury.

The presence of these cells in the fetal circulation (and their availability after delivery for isolation from UCB) is due to their mobilization in response to hypoxia and the increase of pro-mobilizing cytokines in peripheral blood induced by multiple small tissue/organ injuries in the newborn baby during delivery [3, 4]. We envision that mobilization of these cells into newborn peripheral blood could be a kind of autologous physiological stem cell therapy that all people experience in early life.

Interestingly, in contrast to the intense attention that has been paid to stem cells in UCB, studies on the stem cells that are induced in maternal blood are lacking. It is well known that, like the newborn child, the mother is exposed to several types of injuries related to delivery, such as damage due to passage of the fetus through the reproductive tract, hypoxia, and the release of pro-mobilizing cytokines and bioactive lipids [3–5]. To address the possibility of a parallel induction of stem cells in the maternal circulation, we analyzed the number of circulating HSCs, MSCs, EPCs, and VSELs in maternal peripheral blood 12–24 h before and 24 h after delivery and found that changes in circulating stem cells in maternal blood parallel those usually found in UCB.

Based on this finding, we conclude that stem cell mobilization occurs on both sides of the fetal–maternal blood barrier, and most likely the increase in the number of circulating stem cells in maternal blood indicates that they play a role in repairing small tissue/organ injuries that occur in the mother’s body due to delivery. Further studies are needed to determine whether these changes correspond with other clinical parameters, such as the extent of the mother’s internal injuries and the Apgar status of the newborn child, and whether there are changes in the number of circulating stem cells in maternal blood after planned C-sections.

## Materials and Methods

### Study Patients

All women (17) gave written informed consent before enrollment in this project, and all gave natural birth to their children. The age of mothers was 29,5 +/− 4,27 years and they were in a first or second parturition. EDTA-treated peripheral blood was collected twice, before and after natural childbirth. All patient samples were collected with the approval of the Ethics Committee of the Pomeranian Medical University in Szczecin, Poland.

### Flow Cytometry

Peripheral blood (PB) samples were lysed twice using BD Pharm Lyse lysing buffer (BD Bioscience) at room temperature for 10 min and subsequently washed in phosphate-buffered saline (PBS) with 2% fetal bovine serum (FBS; Sigma) to yield total nucleated cells (TNCs) as described [5]. The TNCs were stained in PBS with 2% FBS on ice for 30 min. Cells were subsequently washed, resuspended, and analyzed using a NAVIOS flow cytometer (Beckman Coulter). At least 10^6^ events were acquired from each sample. The absolute numbers of VSELs and the absolute number of white blood cells were calculated (individually for each patient) per 1 ml PB based on the percentage content of these cells as detected by flow cytometry. Kaluza software (Beckman Coulter) was used for analysis.

#### Analysis of Cells Expressing CD34^+^ and CD133^+^ Populations of Lin^−^CD45^−^ Very Small Embryonic-Like Stem Cells (VSELs) and CD133^+^/Lin^−^/CD45^+^ or CD34^+^/Lin^−^/CD45^+^ Cells Enriched for Hematopoietic SCs (HSCs)

TNCs were stained for hematopoietic lineage markers using the following fluorescein isothiocyanate (FITC)-conjugated antibodies (Abs) against human proteins:CD2 (clone RPA-2.10); CD3 (clone UCHT1); CD14 (clone M5E2); CD16 (clone 3G8); CD19 (clone HIB19); CD24 (clone ML5); CD56 (clone NCAM16.2); CD66b (clone G10F5); and CD235a (clone GA-R2) (all from BD Biosciences). The cells were simultaneously stained for the pan-leukocytic marker, CD45, with phycoerythrin (PE)-conjugated Abs (clone HI30; BD Biosciences) and one of the following Abs:allophycocyanin (APC)-conjugated CD34 (clone 581; BD Biosciences) or APC-conjugated CD133 epitope 1 (CD133/1; Miltenyi Biotec). Additionally, the following isotype controls were used:FITC-conjugated mouse IgG1 (clone MOPC-21), mouse IgG2a (clone G155-178), and mouse IgG2b (clone 27–35); PE-conjugated mouse IgG1 (clone MOPC-21); and APC-conjugated mouse IgG1 (clone MOPC-21) (all from BD Biosciences). In addition, the APC-conjugated isotype control mouse IgG1 (clone IS5-21F5; Miltenyi Biotec) was used.

#### Analysis of the CD34^+^/CD133^+^/KDR^+^ Population of Endothelial Progenitor Cells (EPCs) and the CD45^−^/CD105^+^/CD90^+^/CD29^+^ Population of Mesenchymal SCs (MSCs)

Staining for EPCs and MSCs was performed with fluorescence-labeled antibodies for CD45 antigen (FITC; clone HI3, BD Biosciences), CD133 (APC; clone CD133/1, Miltenyi Biotec), CD34 (PE-Cy7; clone 4H11, BD Biosciences), KDR (also known as VEGFR2; PE; clone 89106, R&D Systems), CD105 (APC; clone 43A3, BioLegend), and CD90 (PE clone 5E10, BioLegend).

#### Clonogenic BFU-E and CFU-GM Assays of BM MNCs Isolated from Patients

After RBCs from PB were lysed (Pharm Lyse buffer; BD Biosciences), nucleated cells from hPB were subsequently washed twice and used for BFU-E and CFU-GM assays. The cells were counted and resuspended in human methylcellulose base medium provided by the manufacturer (R&D Systems), supplemented with 25 ng/ml recombinant human granulocyte macrophage colony-stimulating factor (mGM-CSF) and 10 ng/ml recombinant human interleukin 3 (IL-3; Millipore, Billerica, MA, USA). To evaluate the number of clonogenic progenitor cells, BM MNCs were supplemented with erythropoietin (Epo, 5 U/ml; Stem Cell Tech., Vancouver, BC, Canada) plus 5 ng/ml stem cell factor (SCF) and resuspended in methylcellulose base medium (for determining the number of burst-forming units-erythroid). CFU-GM cultures were incubated for 10 days and BFU-E cultures for 14 days (37 °C, 95% humidity, and 5% CO_2_), at which time they were scored under an inverted microscope for the number of each type of colony. Each clonogenic test was performed in duplicate.

#### Statistical Analysis

Arithmetic means and standard deviations were calculated using Statistica 10 software (StatSoft). Statistical significance was defined as *P* < 0.05. Data were analyzed using Student’s t-test.

## Results

### Detection of Mobilized HSCs, VSELs, MSCs, and EPCs in Peripheral Blood of Mothers 24 h After Delivery

**Fig. 1:**
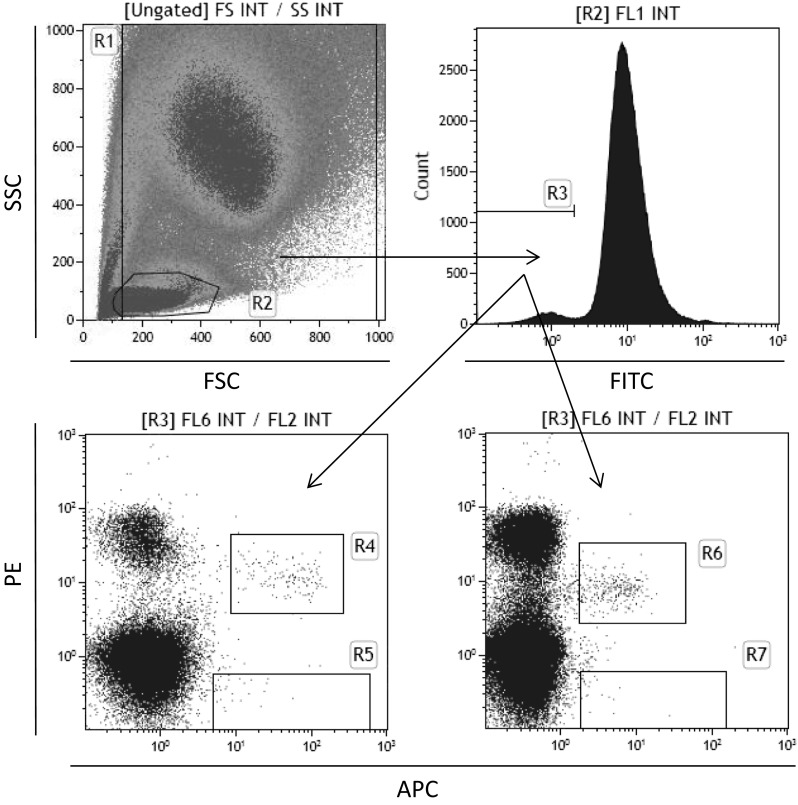
Identification of VSELs in maternal peripheral blood. Gating strategy for analyzing human VSELs in PB. VSELs were identified and enumerated as Lin^−^/CD45^−^/CD34^+^ and Lin^−^/CD45^−^/CD133^+^ cells. hPB-derived TNCs were visualized by a dot-plot based on FSC versus SSC signals. Cells from region R2 were further analyzed for hematopoietic lineage marker expression, and all the Lin^−^ events are included in region R3. The Lin^−^ population was subsequently analyzed based on CD34 and CD45 antigen expression, and two populations of CD34^+^ cells were distinguished based on CD45 expression; that is, Lin^−^/CD45^−^/CD34^+^ (VSELs) in region R5 and Lin^−^/CD45^+^/CD34^+^ (HSPCs) in region R4. Alternatively, the Lin^−^ population was analyzed based on CD133 and CD45 antigen expression, and two populations of CD133^+^ cells were distinguished based on CD45 expression; that is, Lin^−^/CD45^−^/CD133^+^ (VSELs) in region R6 and Lin^−^/CD45^+^/CD133^+^ (HSPCs) in region R7

**Fig. 2:**
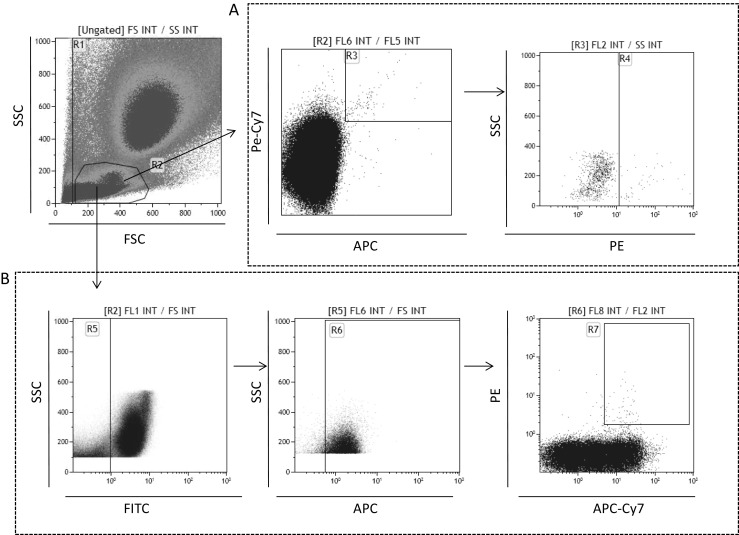
Gating strategy for analyzing human EPCs (**a**) and MSCs (**b**) in maternal peripheal blood. **a** EPCs were identified and enumerated as AC133^+^/CD34^+^ /KDR^+^ cells in region R4. **b** MSCs were identified and enumerated as CD45^−^/CD105^+^/CD29^+^/CD90^+^ cells in region R7

We employed multicolor staining and FACS to analyze the number of circulating HSCs and VSELs (Fig. 1) as well as EPCs and MSCs (Fig. 2) present in maternal peripheral blood 24 h after delivery. By employing a gating strategy to exclude potential artifacts, we were able to detect these cells in maternal peripheral blood. HSCs were analyzed as CD34^+^CD45^+^Lin^−^ cells and some of them as CD133^+^CD45^+^Lin^−^ cells (Fig. 1). This staining strategy allows for identification of primitive Lin^−^ HSCs [5]. At the same time, by focusing on small cells, we were able to detect CD34^+^CD45^−^Lin^−^ and CD133^+^CD45^−^Lin^−^ VSELs (Fig. 1).

Multicolor staining and FACS analysis was also employed to identify EPCs and MSCs (Fig. 2). These cells were identified as CD133^+^CD34^+^KDR^+^ and CD45^−^CD105^+^CD29^+^CD90^+^ cells, respectively [5].

### An Increase in the Number of VSELs, EPCs, and MSCs in Peripheral Blood of Mothers After Delivery

**Fig. 3:**
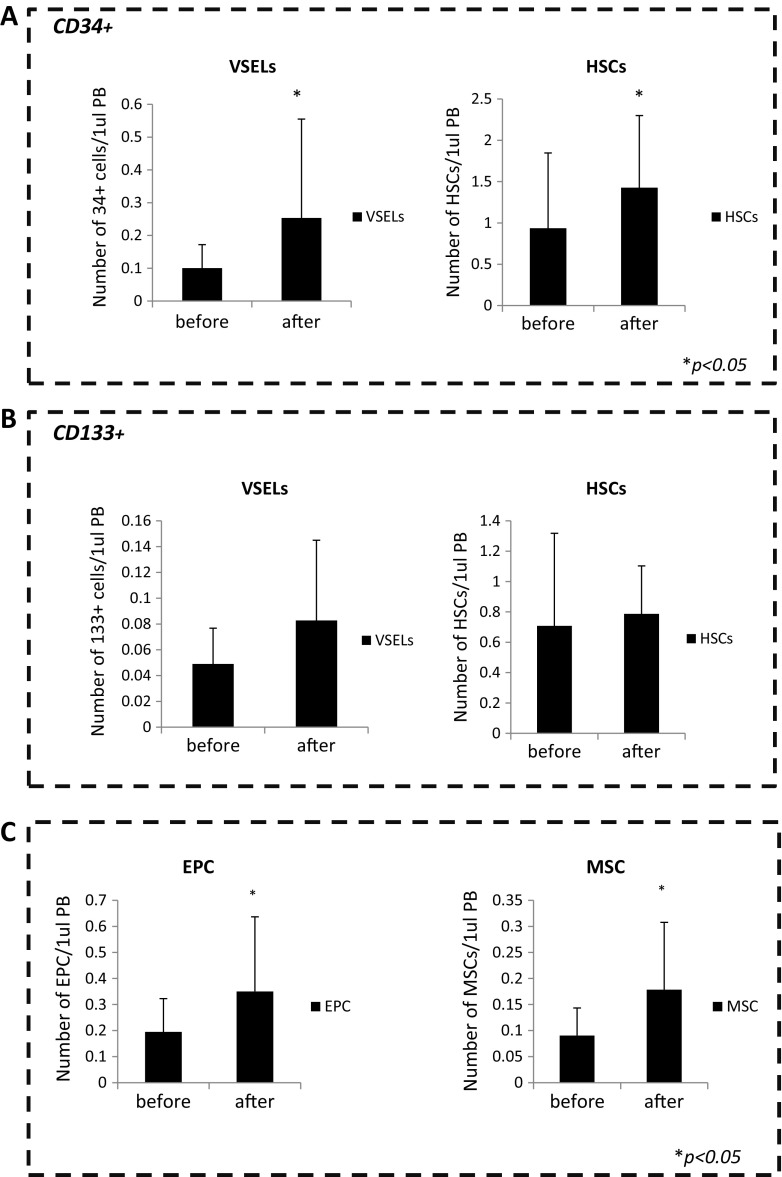
Mobilization of stem cells before and after natural childbirth. **a** Bar graphs showing the absolute numbers of circulating Lin^−^/CD45^−^/CD34^+^ cells (VSELs) and Lin^−^/CD45^+^/CD34^+^ cells (HSCs) in peripheral blood before and after natural childbirth. **b** Bar graphs showing the absolute numbers of circulating Lin^−^/CD45^−^/CD133^+^ cells (VSELs) and Lin^−^/CD45^+^/CD133^+^ cells (HSCs) in peripheral blood before and after natural childbirth. **c** Bar graphs showing the absolute numbers of circulating EPCs and MSCs in peripheral blood before and after natural childbirth

In our analysis we observed an increase in the number of mobilized CD34^+^ VSELs and HSCs in mothers following delivery (Fig. 3a). The number of these cells circulating in PB was significantly increased compared with their numbers in peripheral blood before delivery. We also observed an increase in the number of CD133^+^ VSELs and HSCs in maternal peripheral blood after delivery, although this increase was not statistically significant (Fig. 3b). At the same time, we found a significant increase in the numbers of EPCs and MSCs 24 h after delivery (Fig. 3b).

### An Increase in the Number of Clonogenic Progenitors in PB in Mothers After Delivery

**Fig. 4:**
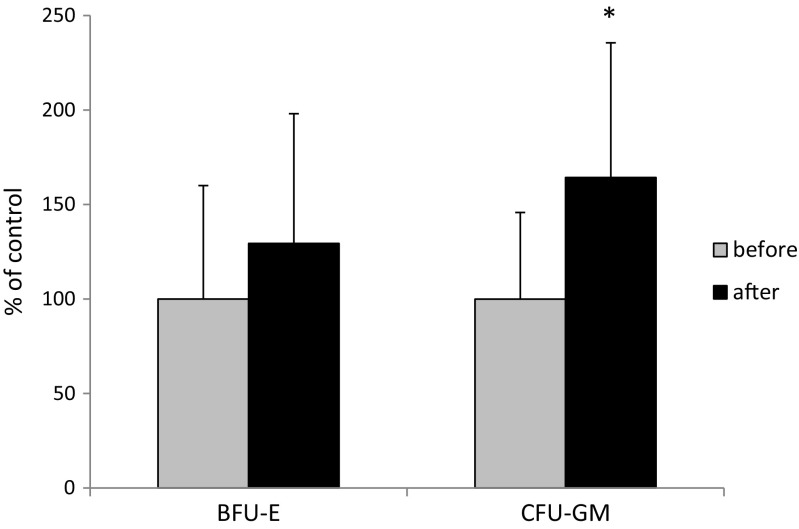
In vitro assay for BFU-E and CFU-GM clonogenic progenitor cells in hPB. Blood from women before (*n* = 17) and after (*n* = 10) delivery was collected and evaluated by the number of BFU-E and CFU-GM cells. Results from two separate experiments are pooled together. After 10 and 14 days following delivery, there were increases in the numbers of BFU-E and CFU-GM progenitor cells circulating in the peripheral blood of the women, *p* ≤ 0.05

Finally, we became interested in the increase in the number of clonogenic progenitors for the erythroid and myeloid lineages. As shown in Fig. 4, while the number of clonogenic progenitors for the erythroid lineage did not change significantly, there was an increase in the number of progenitors for the granulocyte-myeloid lineage (Fig. 4).

## Discussion

The most important observation of this paper is that stress related to delivery mobilizes several types of stem cells, including HSCs, MSCs, EPCs, and VSELs, into maternal blood, which demonstrates that an increase in the number of circulating stem cells occurs on both sides of the fetal–maternal circulation barrier.

It is well known that HSCs circulate under steady-state conditions at detectable levels in PB, with their numbers increasing in response to inflammation, stress, and tissue or organ injury [5–9]. In parallel to HSCs, other rare types of non-hematopoietic stem cells also circulate in PB. Moreover, under normal steady-state conditions the circulation of HSCs is regulated in humans by a circadian rhythm, with the peak in the number of circulating stem cells occurring in the early morning hours and the nadir at night [10]. This increase correlates with the occurrence of deep sleep hypoxia at night and activation of the complement cascade as well as changes in tonus of BM sympathetic innervation [10, 11].

Stem cells are released during stress situations from their niches, mainly from BM into PB, by a cascade of events that activates the abovementioned complement cascade, whose cleavage fragments (e.g., C5a and _desArg_C5a) promote the release of several proteolytic [12] and lipolytic [13] enzymes from BM-residing granulocytes and monocytes. These enzymes cooperate together and attenuate interactions between, on the one hand, the alpha chemokine stromal-derived factor 1 (SDF-1) and the CXCR4 receptor and, on the other hand, vascular adhesion molecule 1 (VCAM-1) and very late antigen-4 VLA4 integrin, which are the most important retention axes for stem cells in BM.

An important function of the stem cell circulation is for these cells to travel through the blood stream and enter different tissues and organs to maintain anti-inflammatory or pro-regenerative homeostasis of the body [8, 14–16]. Accordingly, when pathogens are present in peripheral tissues, circulating HSCs may differentiate into cells involved in local tissue defense (e.g., granulocytes, monocytes, or dendritic cells) [16]. A similar role in patrolling peripheral blood is played by other types of stem cells, such as, MSCs, EPCs, and VSELs, with their number increasing in various stress situations related to tissue and organ damage, such as heart infarct, stroke, skin burns, or intestinal inflammation [6, 8, 17, 18].

One of the biggest stresses in life is childbirth, which is connected with various degrees of tissue/organ damage, and this trauma may explain why UCB is enriched in circulating stem cells [8, 19]. We proposed in the past that this could be a physiological process involving a natural stem cell therapy in which circulating stem cells repair small tissue/organ injuries that occur during delivery [5].

With this in mind, it is somewhat surprising that there has been no extensive investigation to determine whether delivery stress and tissue/organ injury in the mother also triggers mobilization of stem cells into the circulation. We report here for the first time that, 24 h after delivery, there is a statistically significant increase in the number of CD34^+^ VSELs and HSCs as well as MSCs and EPCs circulating in PB. We also observed an increase in the number of mobilized CD133^+^ VSELs, although this was not statistically significant. All these results together support the concept that stem cells are released into maternal PB after delivery and have a role in repairing tissue/organ injuries to the mother’s body.

We are also aware of some limitations in our study. First of all, further research is needed to measure the kinetics of changes in the number of circulating stem cells after delivery, as the 24-h “post partum” point selected by us may be not optimal. Secondly, it is important to correlate the release of stem cells with the clinical status of the mother and the Apgar status of the newborn child. Moreover, it would be interesting to perform similar studies after planned C-sections. It is also important to measure the level of factors in maternal blood that are crucial for egress of different types of stem cells into PB, such as the bioactive lipids sphingosine-1-phosphate (S1P) and ceramide-1-phosphate (C1P), stromal-derived factor 1 (SDF-1), or vascular endothelial growth factor (VEGF) [20–22]. On the other hand, since activation of the complement cascade triggers the release of stem cells from their niches, it is important to measure the level of C3 and C5 cleavage fragments that occur during complement cascade activation [23, 24]. Finally, it would be interesting to see whether the level of mobilized stem cells correlates with the recovery status of the women after delivery. Further work is also needed to assess a role of various stem cells including MSCs, EPCs and VSELs in regeneration of maternal tissues damaged during delivery process [8, 16, 25–27].
